# The Present and Future of the Clinical Use of Physiological Traits for the Treatment of Patients with OSA: A Narrative Review

**DOI:** 10.3390/jcm13061636

**Published:** 2024-03-13

**Authors:** Yvonne Chu, Andrey Zinchuk

**Affiliations:** Section of Pulmonary, Critical Care and Sleep Medicine, Department of Internal Medicine, Yale School of Medicine, 300 Cedar Street, The Anlyan Center, 455SE, New Haven, CT 06519, USA; yvonne.chu@yale.edu

**Keywords:** OSA traits, upper airway collapsibility, loop gain, airway muscle responsiveness, arousal threshold, OSA therapy

## Abstract

People with obstructive sleep apnea (OSA) are a heterogeneous group. While many succeed in the treatment of their OSA, many others struggle with therapy. Herein, we discuss how anatomical and physiological factors that cause sleep apnea (OSA traits) impact treatment response and may offer an avenue for more precise care. These OSA traits, including anatomical (upper-airway collapsibility) and physiological (loop gain, airway muscle responsiveness, and arousal threshold) factors, may help determine who can succeed with continuous positive airway pressure, oral appliances, hypoglossal nerve stimulation, or pharmacotherapy. In the future, identifying OSA traits before initiating treatment may help guide the selection of the most effective and tolerable therapy modalities for each individual.

## 1. Introduction

Obstructive sleep apnea (OSA) is estimated to affect 1 billion adults aged 30–69 years worldwide [1], with prevalence increasing 2–3-fold in older adults (>65 years of age) compared to middle-aged adults [2,3]. Untreated OSA is associated with major causes of morbidity and mortality, including hypertension, strokes, coronary artery disease, metabolic syndrome, cognitive impairment, and mood disorders [4,5,6]. The timely diagnosis and treatment of OSA may have a role in mitigating the development or progression of these comorbid conditions [7]. Positive airway pressure (PAP) therapy is the gold standard and most efficacious therapy for OSA [8], and is prescribed to 80% of those diagnosed with OSA [9].

While PAP can eliminate airway obstruction in many patients, this “one size fits all” approach of PAP therapy for OSA has limitations. Long-term adherence to continuous positive airway pressure (CPAP) therapy is poor and varies widely. For example, adherence ranges from 50% in women aged 18–30 years to 80% in men aged 71–80 years [10]. Individuals from disadvantaged socioeconomic backgrounds struggle with PAP therapy even more (40% adherence) [11]. Numerous reasons for poor adherence have been identified, including claustrophobia, pressure intolerance, low self-efficacy or motivation for PAP use, and insomnia [12,13]. Despite advancements in PAP device technology, such as new masks and behavioral interventions, PAP adherence has not improved over the past 20 years [12,14]. Moreover, PAP therapy may not always be efficacious, with clinical cohorts and trials revealing that 25–30% of those using PAP therapy have a residual apnea–hypopnea index (AHI) score of >10/h [15,16]. Finally, patients do not consistently experience symptomatic relief with PAP therapy, and the lack of perceived symptomatic improvement is linked to poor PAP adherence [17]. As a result, many patients enter cycles of PAP trial and failure followed by attempts at non-PAP treatments [18]. This is, in part, because currently, there are no standardized methods to predict who will respond to PAP therapy and who will benefit from non-PAP therapies. New approaches in therapy decision-making are needed.

Emerging research shows that OSA is a heterogeneous disorder in terms of causes, presenting symptoms, and consequences [19,20,21]. Proposed models of susceptibility to OSA suggest that in addition to established anatomic causes (e.g., obesity, nasal obstruction, craniofacial structure, hyoid bone positioning, and upper airway edema), physiologic traits also predispose individuals to OSA. Tailoring OSA therapy based on the causes of sleep apnea in each individual has been proposed as a promising approach to precision medicine in OSA [21]. The overview of potential approaches to precision medicine in OSA, including factors beyond OSA’s physiological traits, is highlighted in other reports [22,23,24].

The purpose of this manuscript is to review the anatomical and physiological contributors to OSA, describe the methods for their assessment, discuss potential applications based on current evidence, and highlight key obstacles in developing precision medicine approaches to OSA based on these traits. The key points of this manuscript are summarized in Table 1. 

## 2. OSA Traits

Four key traits contribute to the development of OSA [25]. These include increased upper-airway collapsibility, poor upper-airway dilator-muscle responsiveness, ventilatory-control instability (high loop gain), and a low arousal threshold. Different combinations and varying degrees of these traits may cause OSA in each individual [26].

**Figure 1:**
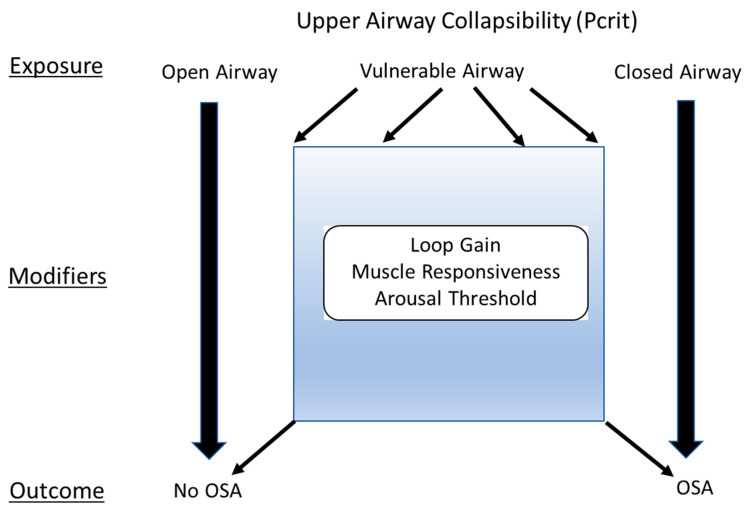
The interplay of the four key traits in predicting the outcome of OSA. In this conceptual framework, the upper airway collapsibility, measured by the critical closing pressure (P_crit_) is the exposure. A very negative P_crit_ means the airway is non-collapsible (or an “open airway”), which results in no OSA phenotype. A high P_crit_, on the other hand, means the airway is highly collapsible (or a “closed airway”) and will inevitably result in the OSA phenotype. In between is the vulnerable airway, in which the modifying traits (loop gain, muscle responsiveness, and arousal threshold) can influence the phenotypic outcome of OSA vs. no OSA. The conceptual framework is adapted from Owens et al., Sleep 2015; 38: 961–70 [26].

One framework to understand how the four traits cause OSA examines upper-airway collapsibility (a surrogate of anatomic predisposition to collapse) as the key exposure (Figure 1). An upper airway that is not collapsible (i.e., an “open” airway) will result in the outcome of no OSA. In contrast, a highly collapsible airway (i.e., a “closed” airway) will consistently lead to OSA. For individuals with a “vulnerable” upper-airway anatomy (collapsibility between the “open” and “closed” airways), developing OSA (or no OSA) may depend on the modifying effects of the remaining physiological traits (loop gain, muscle responsiveness, arousal threshold) [26]. 

### 2.1. Anatomic Contributions to Upper-Airway (UA) Collapsibility (the Exposure)

The concept of upper-airway collapsibility comes from the Starling resistor model, in which the airway “tube” spans from the posterior aspect of the nasal septum to the larynx [27,28]. This tube (airway) is susceptible to collapse because it has no rigid support. When the pressure surrounding the tube exceeds the pressure within the tube, collapse occurs. The critical closing pressure (P_crit_) is the pressure within the tube equal to the surrounding pressure, and any increase in surrounding pressure beyond the P_crit_ results in collapse. The more negative the P_crit_, the less collapsible the airway. For example, a P_crit_ of −5 cm water suggests a non-collapsible UA, while a P_crit_ of 5 cm water suggests a highly collapsible UA.

Factors that determine UA collapsibility are those that modify the airway diameter. These include obesity, which thickens the pharyngeal walls, and neck flexion, which also narrows the airway [29,30]. Similarly, low lung volume from abdominal obesity reduces tracheal traction, which is needed to unfold the airway and stiffen its walls [31]. Moving from the supine to lateral position results in a less collapsible airway and a lower P_crit_ [32]. Gender differences in airway collapsibility exist. Women have a less collapsible airway than BMI-matched men, in part due to having shorter airways (less opportunity for collapse) and a smaller cross-sectional area of the soft palate [33,34]. 

### 2.2. Physiological Contributors to OSA (the Effect Modifiers)

#### 2.2.1. Ventilator Control Instability (High Loop Gain)

**Figure 2:**
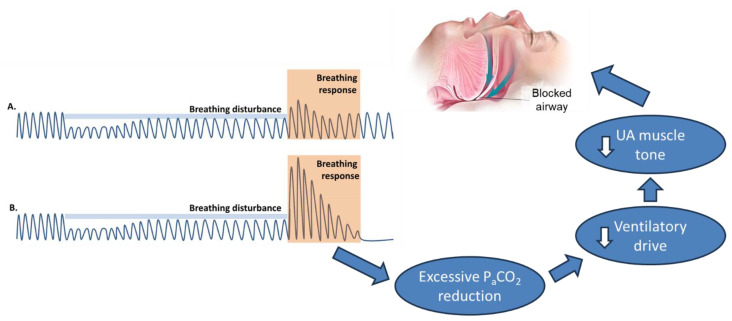
The pathogenesis of high-loop-gain OSA. In individuals with normal loop gain (e.g., flow tracing in (**A**)), the decrease in ventilation (hypopnea—“breathing disturbance” denoted as a blue bar) leads to an expected ventilatory response that is driven by the individual’s chemosensitivity to PaCO_2_ changes (a small rise in flow after the “breathing disturbance” is removed), before return to normal ventilation. Pathology arises in individuals with high loop gain (e.g., flow tracing in (**B**)); the same decrease in ventilation (hypopnea) leads to an exaggerated ventilatory response (hyperpnea) due to heightened chemosensitivity to PaCO_2_ changes, resulting in the cycling between hypopneas/apneas and hyperpneas.

The respiratory system is composed of the ventilatory controller (chemoreceptors) and the ventilatory pump (the UA and the lungs) that are connected by a feedback loop (circulation). The arterial carbon dioxide level (PaCO_2_) is a key determinant of ventilatory drive produced by the controller. In OSA, the decrease in ventilation (e.g., hypopnea) leads to a response (e.g., hyperpnea), the magnitude of which is determined by the individual’s chemosensitivity to PaCO_2_ changes. Because ventilatory drive affects not only the thoracic pump muscles (e.g., the diaphragm) but also the UA muscles [35], excessive PaCO_2_ reductions from hyperpnea can result in airway collapse and obstructive events [36]. Thus, in individuals with OSA who have an overly sensitive ventilatory drive, the respiratory system can be unstable, cycling between hypopneas and hyperpneas [37] (Figure 2). Loop gain (LG) is a term describing how strongly a feedback loop system (i.e., respiratory system) responds to a disturbance. High LG represents ventilatory instability [37] and is considered an etiologic factor in one-third of patients with OSA [25].

#### 2.2.2. Pharyngeal Muscle Responsiveness

The upper-airway dilator muscles keep the upper airway patent, with the most studied dilator being the genioglossus. Muscle activity of the genioglossus declines from wake to sleep and furthermore from N2/N3 to REM sleep [38]. This decline may be the mechanism for REM-dependent OSA, especially in those who rely on the genioglossus to maintain airway patency during REM sleep [39]. While poor muscle responsiveness can result in OSA, vigorous muscle responsiveness may protect individuals from OSA. For example, in a study examining upper-airway muscle responsiveness (UAMR) between overweight/obese individuals with and without sleep apnea, the UAMR was 3-fold higher in those with obesity and no OSA [40], suggesting a protective effect. Progesterone increases genioglossus activity and dilates the airway [41]. Reduced levels of progesterone post-menopause may play a role in the pathogenesis of OSA in women.

#### 2.2.3. Low Arousal Threshold

Arousal threshold (ArTH) measures the propensity to awaken from a respiratory stimulus (e.g., apnea). Arousals are necessary to reopen the UA and terminate obstructive events in some individuals [42,43]. A low ArTH (too easy of an arousability), however, has been postulated to lead to OSA [25]. This is partly because easy arousability may lead to frequent, short respiratory events. The resulting sleep fragmentation lowers the propensity for deeper, N3 sleep, during which sufficient ventilatory drive to the UA muscles may open the airway before a frank apnea or hypopnea develops. Similarly, ventilatory overshoot during arousal’s opening of the UA lowers the PaCO_2_ (and thus ventilatory stimuli for airway opening), promoting the recurrence of UA collapse [42]. A low ArTH is more common in individuals with REM-dependent OSA [44], and among those who are non-obese, older, and taking antidepressants [45]. Individuals with post-traumatic stress disorder may also have a lower ArTH, presumably due to their hyperadrenergic state [46]. 

#### 2.2.4. Sex and Race Differences in Pathophysiology of OSA

A recent analysis of a diverse, multi-community cohort (Multi-Ethnic Study of Atherosclerosis, N = 1971; age range, 54–93 years) suggests that each of the four traits contribute differently to the pathophysiology of OSA in each sex and race/ethnicity [47]. For example, both increased UA collapsibility and reduced airway muscle responsiveness account for the majority of differences in AHI scores between males and females. Compared to white individuals with OSA, Black individuals exhibit lower AHI scores, potentially due to lower UA collapsibility despite having a higher LG. In contrast, UA collapsibility alone explained almost 90% of the differences in AHI scores between white individuals and individuals of Chinese ancestry, with adjustment for obesity.

Despite lower BMI rates among Asians, the prevalence of OSA is similar compared to Caucasian cohorts. Until recently, and as noted above, this has been attributed to greater craniofacial restriction (more predisposing anatomy) among Asian individuals [48]. Another analysis examined the role of the ArTH in OSA pathogenesis, comparing Caucasian (*n* = 163) and Chinese (*n* = 185) patients with OSA [49]. A low ArTH was a less common pathophysiological mechanism (28% vs. 49%) among Chinese versus Caucasian individuals with moderate-severe OSA, especially among those with mild craniofacial-anatomical restriction. In sum, findings from such studies suggest that OSA mechanisms vary across sex and race, which should be considered as investigators to assess the role of the traits in precision medicine in OSA. 

#### 2.2.5. Role of Comorbid Conditions in the Pathophysiology of OSA

Little data exist regarding the contribution of physiological traits to OSA in those with comorbid conditions. In a small study (*n* = 10) of non-hypercapnic chronic obstructive pulmonary disease (COPD) and OSA, UA collapsibility played a key role in OSA in only two individuals. The majority exhibited a high LG or a low ArTH, which were inversely correlated with markers of air trapping (high residual volume and residual volume to total lung capacity ratio) [50], suggesting that the worse the COPD severity, the lower the ArTH, resulting in fragmented sleep. Notably, ventilatory drive is reduced in REM sleep [51] and may explain the worsening of OSA in COPD overlap syndrome. In those with comorbid insomnia and OSA (COMISA), the UA is less collapsible and the ArTH is lower compared to those with OSA alone [52]. Notably, a low ArTH contributed to UA collapsibility in patients with COMISA only, and not those with OSA alone. Treating the underlying chronic insomnia in these patients may be key to the treatment of COMISA. In veterans with comorbid post-traumatic stress disorder (PTSD) and OSA, the presence of a low ArTH and insomnia predicted poor CPAP utilization, while a low ArTH alone was not a predictor [53]. More research is needed to assess the role of physiological traits in the pathogenesis of OSA and their impact on therapy selection among individuals with these and other comorbid conditions.

## 3. Measurement of OSA Traits

**Figure 3:**
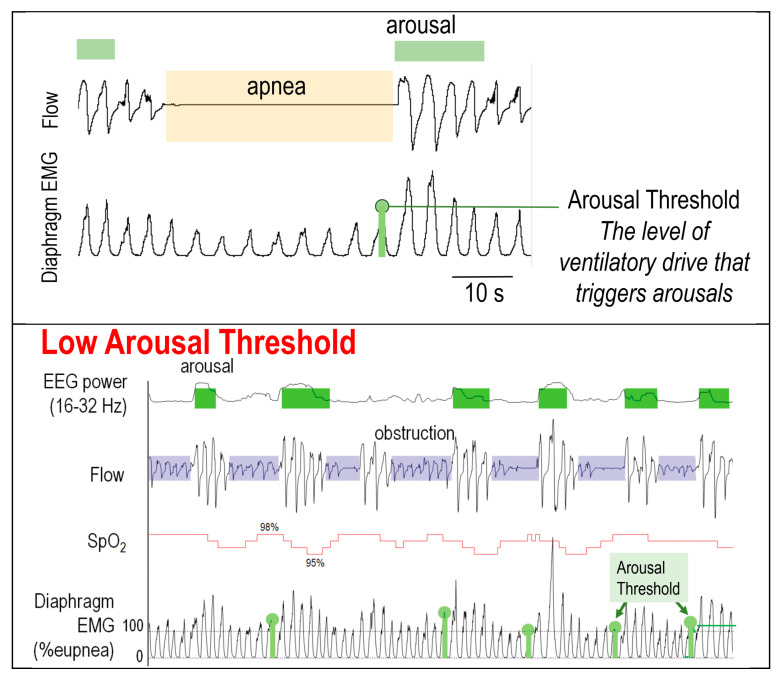

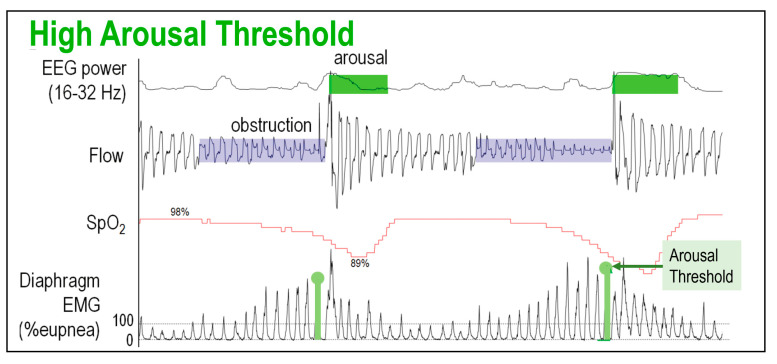
(**Top**) Determining ArTH from research polysomnography. Note that ventilator drive determined from the diaphragm EMG signal can be replaced by ventilatory drive (V_drive_) from routine clinical polysomnography. OSA with low (**Middle**) and high (**Bottom**) ArTH. Note low ventilatory drive required for arousal (short vertical green bars within the diaphragm EMG signal), frequent respiratory events (purple), and minimal desaturations (red line) in low ArTH OSA in contrast to higher drive needed for and arousal (high vertical green bars within the diaphragm EMG signal), and less frequent and deeper obstructive events in high ArTH OSA. Images courtesy of Drs. Scott Sands and Laura Gell; top figure concept adapted from Gell LK et al. [61] Thorax 2022; 77: 707–716 online-supplement.

The gold standard for measuring physiologic OSA traits is invasive and is elegantly described by Eckert [54]. In brief, the measurement of upper-airway collapsibility, or the critical closing pressure (P_crit_), involves the use of a pneumotachometer to assess flow and an esophageal pressure catheter [55] or a diaphragmatic electromyography (EMG) to assess ventilatory drive throughout the night during polysomnography with rapid CPAP pressure changes [56]. The LG and ArTH are determined from the ventilatory drive and responses to flow disturbances (CPAP pressure drops). For example, the ArTH reflects a median ventilatory drive just before an arousal from a series of CPAP drops (see Figure 3). Similarly, airway dilatory muscle responsiveness measurements require a surface EMG [57] of the tongue. Such methods are not practical outside of physiological research studies.

Advances in signal processing have enabled the development of an automated, non-invasive method for measuring OSA traits from a diagnostic PSG [58,59,60]. In brief, an in-laboratory polysomnogram is segmented into 7 min windows of non-REM sleep. The nasal pressure signal during a 7 min window is used to estimate the “baseline” eupneic non-obstructed ventilation (ventilatory drive (V_drive_)). LG is quantified as the V_drive_ response to ventilatory disturbance (e.g., hypopnea). The arousal threshold (ArTH) is calculated as the V_drive_ immediately preceding an arousal. To determine UA collapsibility (1/V_passive_), a plot of the breath-by-breath values of ventilation and V_drive_ for NREM sleep is generated, and V_passive_ is calculated as ventilation at the eupneic V_drive_. Lower V_passive_ values represent greater collapsibility (i.e., a higher P_crit_). Pharyngeal muscle compensation (V_comp_) is the difference between ventilation at an elevated V_drive_ (precisely, at the ArTH) and V_passive_. The advantage of this approach is that it enables the measurement of OSA traits from a clinical polysomnography. This has led to an exponential growth in studies examining the traits and outcomes of OSA therapy. While promising, the method requires an understanding of signal processing and specialized arousal scoring, limiting its widespread use in clinical outcomes research.

## 4. Point of Care Surrogate Measures of the Traits 

Point of care (POC) tools based on routine clinical sleep study metrics to estimate the four OSA traits [62,63,64,65] have been developed. For example, Edwards et al. demonstrated that the therapeutic CPAP level on a titration study can discriminate a mildly from a moderately/severe collapsible UA. A therapeutic CPAP requirement of ≤8 cm of water was 89% sensitive and 84% specific for detecting a mildly collapsible airway [62].

The same group also showed that a simple clinical score can predict a low ArTH. A point is given for each: an apnea–hypopnea index (AHI) score of <30 events per hour, nadir oxygen saturation as measured by pulse oximetry > 82.5%), and the fraction of the AHI that are hypopneas (F_hypopneas_) > 58.3%. A score of two or more predicts a low ArTH with 80% sensitivity and 88% specificity [65]. 

Methods to estimate LG from the AHI and from F_hypopneas_ are less accurate [63]. LG can be effectively estimated using the formula LG = 2π/[2πDR – sin(2πDR)] in those with treatment-emergent central sleep apnea (TE-CSA) [64]. Ten periodic breathing cycles with optimal CPAP during a titration study are needed. The DR is the ratio of the duration of the ventilatory phase (time from the end of one apnea to the start of the next) to the total cycle duration (time from the end of one apnea to the end of the next). 

Dutta et al., developed a decision tree prediction model using standard metrics (e.g., AHI, REM AHI, and BMI) to predict the “good”, “moderate”, and “bad” levels of the four OSA traits [66]. If such tools are made user-friendly and are validated, they may offer a way for clinicians to derive these traits with clinical data. 

Pattern recognition may also be useful when assessing ventilatory stability. For example, in contrast to the V-shaped pattern of oxygen desaturation seen in REM-related OSA, the “zipper-like” pattern of oxygen desaturation seen on hypnograms can be more consistent with the periodic breathing seen in NREM-predominant OSA, which is more likely to exhibit high LG [67,68]. 

In summary, while several promising POC tools to estimate the OSA traits exist (Table 2), they are limited by low accuracy, dependence on OSA severity (e.g., AHI score), or the requirement of PAP titration in the sleep laboratory. As with the promising non-invasive, automated methods to measure these traits [58,60], these tools also require prospective validation. 

## 5. Precision Treatment of OSA Using the Traits

**Figure 4:**
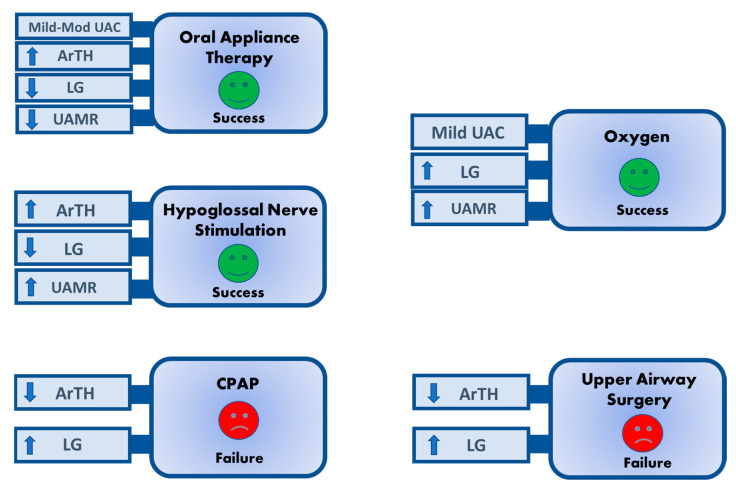
The traits predicting OSA treatment success and failure. Current evidence supporting the use of OSA traits to predict treatment response is summarized here. The identification of patients at risk of failure/success early on may lead to closer monitoring of the therapy and aid in the counseling of patients. ArTH = arousal threshold; LG = loop gain; UAC = upper-airway collapsibility; UAMR = upper-airway muscle responsiveness; up arrow = increased; down arrow = decreased.

Here we will discuss the application of the OSA traits to improve the precision of OSA therapy approaches, in the order of most to least supported by available evidence: (1) predicting responses to established OSA therapies (see Figure 4), (2) guiding multi-modal therapy for OSA, and (3) targeting the traits to select initial therapy for OSA. 

### 5.1. Use of Traits to Predict Responses to OSA Therapy

Most of the literature on the OSA traits and OSA therapy focuses on predicting treatment response. OSA traits may help identify patients at risk of failure of a given treatment early on, prompting the provider to monitor treatment more closely and be prepared to use alternative or adjunctive therapies. 

#### 5.1.1. Conventional PAP therapy

CPAP and bilevel PAP therapy are considered conventional forms of PAP therapy. When assessed by the POC tool, a low ArTH was associated with PAP non-adherence (OR 4.4) at 3 months in a sleep clinic cohort [69]. Among non-obese veterans with OSA, a low ArTH (assessed by the POC tool) was also associated with a 45% reduction in long-term use of CPAP therapy. Such findings are consistent across populations and the measurements of OSA traits from polysomnography. In post-stroke patients, a reduction in ArTH of 8% and an increase in UA responsiveness of 33% were associated with a 1 h reduction in CPAP use [70]. Similarly, in patients with coronary artery disease and OSA, a phenotype of low ArTH and extremes of UA muscle responsiveness were associated with an over 2 h/night lower CPAP adherence [71]. In sum, close monitoring is prudent in those with a low ArTH during conventional PAP therapy. If PAP intolerance develops, interventions to improve adherence may be of benefit, including motivational interviewing [72,73], CBTi in those with comorbid insomnia [74], or a short course of a sedative hypnotic [75]. 

In cases of treatment emergent central sleep apnea, a high LG (>2) may predict persistence of TE-CSA in response to conventional PAP therapy at 1 month [64]. This may be considered when deciding on adaptive servo-ventilation (ASV) at the start of PAP therapy in those with TE-CSA. 

#### 5.1.2. Oral Appliance

Oral appliance therapy (OAT), most commonly the mandibular advancement device, can be an effective and tolerable OSA treatment. Several investigations have identified OSA traits that predict patient response to OAT. In a randomized cross-over study, those with mild UA airway collapsibility and a low/normal LG exhibited the highest AHI reductions with OAT [76,77]. In the largest study of 93 patients with AHI scores of ≥20/h, a lower LG, higher ArTH, moderate (non-mild and non-severe) UA collapsibility, and weaker dilator muscle compensation [78] predicted a greater AHI reduction. A model of OAT responders (AHI ≥ 50%) using these traits exhibited a positive predictive value (PPV) of 83% and a negative predictive value (NPV) of 58%. Notably, none of the clinical parameters, including age, BMI, sex, neck circumference, and REM/NREM AHI, were associated with a change in AHI score with OAT treatment. In summary, those with mild-to-moderate airway collapsibility, lower LG, higher ArTH, and weaker muscle compensation may be better candidates for OAT. 

#### 5.1.3. Upper Airway Surgery

UA collapsibility is reduced by surgery, including revised uvulopalatopharyngoplasty with uvula preservation, with or without concomitant transpalatal advancement (TA), pharyngoplasty, genioglossus advancement, and hyoid suspension. A high LG predicts the failure of upper airway surgery to achieve a ≥50% reduction in and <10/h post-surgical AHI [79]. Similarly, a low ArTH is associated with surgical failure [80]. Despite potential surgical improvements in UA collapsibility, the remaining abnormalities in LG and ArTH are not modifiable by surgery. Therefore, those with a high LG and/or low ArTH may be at high risk of residual OSA with upper airway surgery. 

#### 5.1.4. Hypoglossal Nerve Stimulation

Hypoglossal nerve stimulation is a rapidly growing option for individuals who are intolerant of CPAP therapy. HNS uses a cuff, implanted around the branches of the hypoglossal nerve, to stimulate the genioglossus, protruding it with each breath. Despite stringent selection criteria for this therapy (e.g., BMI < 32 kg/m^2^, lack of complete concentric collapse on sleep endoscopy), about a third of those who are implanted achieve a <50% reduction in and a residual AHI score of ≥20/h [81]. A secondary analysis of the STAR trial of HNS revealed that a high ArTH, low LG, and increased UA muscle responsiveness [82] were associated responses to HNS. Notably, the trait relationships were complex. For example, the LG and ArTH were associated with HNS response in those with mild UA collapsibility. A multivariable prediction model of HNS responders using the OSA traits was better at ruling in success than avoiding failure (PPV and NPV of 83% and 61%, respectively). 

#### 5.1.5. Pharmacotherapy

The purpose of the below section is to provide a context of how pharmacotherapy may affect the OSA traits, rather than a comprehensive review of pharmacotherapy in OSA; this can be found elsewhere [83,84]. Notably, most pharmacotherapy studies do not target individuals based on OSA traits, but use OSA severity cut-offs. In addition to these studies, we highlight a few that select patients based on OSA traits.

Supplemental oxygen is a potential adjunctive therapy for OSA. In a physiologic study of six individuals with OSA, oxygen reduced both the LG and AHI score in those with a high LG, but not in those with a low LG [85]. In a larger (*n* = 36) single-night crossover RCT of oxygen for OSA, a high LG alone did not predict the response to oxygen [86]. However, a high LG in those with mild UA collapsibility and higher UA responsiveness were predictors of oxygen’s success (≥50% AHI reduction). 

The commonly prescribed sleep aid non-benzodiazepine GABA receptor agonists (“z-drugs”), specifically zolpidem 10 mg and eszopiclone 3 mg, appear to increase the ArTH in those with a low ArTH without impacting airway dilator muscle responsiveness, prolonging respiratory events or worsening hypoxemia [87,88]. A longer term, 1-month RCT of 7.5 mg zopiclone (a stereo-isomer of eszopiclone) in patients with OSA showed no effects on hypoxemia or sleepiness or driving simulator performance [89]. The effects on the AHI were inconsistent in these single-night physiologic studies and showed no changes in the 1-month trial [87,88,89]. While there is little effect on the AHI, sedative hypnotics may improve adherence to CPAP therapy [75], potentially by raising the ArTH.

Acetazolamide, a carbonic anhydrase inhibitor, has been studied in both OSA and CSA. Acetazolamide causes urinary excretion of bicarbonates with a metabolic acidosis, leading to respiratory compensation by way of increased ventilation. Notably, at this state, the efficiency of CO_2_ excretion in the lungs is lower (i.e., acetazolamide lowers LG), which stabilizes breathing [90]. A systematic review that included 28 studies and 542 participants found that acetazolamide reduced AHI scores by 38% or 14/h in those with OSA or CSA compared to the controls. It also improved the SpO_2_ nadir by 4 percent [91]. A dose of acetazolamide 500 mg twice daily was shown to reduce LG (with an interquartile range reduction from 2.4–5.4 to 1.4–3.5) [92] and reduce the NREM AHI (from 50/h to 24/h) in those with OSA. Acetazolamide had no significant effect on UA collapsibility, responsiveness or ArTH. Notably, baseline LG alone did not predict a response to acetazolamide [93]. 

Medications have been studied for their potential role in increasing UAMR. Increasing the endogenous levels of norepinephrine in a rat model showed increases in genioglossus muscle activity during NREM sleep [94]. In another study, a muscarinic receptor antagonist disinhibited hypoglossal motor neuron activity during REM sleep [95]. Therefore, upregulating norepinephrine activity during NREM and antimuscarinic activity in REM sleep exhibits potential to increase genioglossus muscle responsiveness throughout sleep. The combination of atomoxetine, a norepinephrine reuptake inhibitor, and oxybutynin, an antimuscarinic, has been studied in a randomized, placebo-controlled, double-blind, crossover trial comparing one night of 80 mg atomoxetine plus 5 mg oxybutynin (ato–oxy) to a placebo. Ato-oxy reduced AHI scores by 63%, increased genioglossus responsiveness 3-fold, and improved hypoxia [96]. While ato-oxy improved UA collapsibility and UA muscle responsiveness in a secondary analysis of this RCT, baseline traits did not predict a response to ato-oxy. In multivariate analyses, only the baseline AHI and F_hypopneas_ predicted a response to ato-oxy [97]. A more recent, larger (*n* = 211), 4-week trial of atomoxetine and aroxybutynin showed a smaller but meaningful effect size (43% AHI reduction) [98]. Other combinations of noradrenergic-antimuscarinic therapies have been proposed and have undergone small trials, as nicely reviewed by Perger and colleagues [83]. They included reboxetine and oxybutynin, which demonstrated an improvement in UAMR and a 59% reduction in AHI scores at 1 week [99]. 

Dual orexin receptor antagonists (DORAs) are an emerging class of medications used to treat insomnia. The combination of atomoxetine-lemborexant has been studied in a small trial of 15 individuals with moderately collapsible upper airways. This combination did not significantly reduce the AHI [100]. Studies are needed to determine the potential role of DORAs in affecting the non-anatomical physiological traits and impact on OSA. 

Precaution should be taken for the use of these pharmacologic agents in the growing population of older adults living with the OSA. They may be at a higher risk for adverse side effects. For example, Z-drugs may increase the risk of gait instability, falls, and fractures in older adults [101,102,103]. Acetazolamide may result in moderate to severe metabolic acidosis in older adults [104]. In an atomoxetine-oxybutynin combination, oxybutynin may increase the risk of delirium [105].

### 5.2. Use of Traits to Guide Multi-Modal Therapy for OSA

Multi-modal therapy is a cornerstone of the management of other chronic disorders (e.g., hypertension and diabetes mellitus), and the management of OSA is likely to be similar. One approach to multi-modal therapy may include addressing anatomic predisposition to OSA (UA collapsibility) alongside adjunctive or rescue therapies targeting physiological traits to improve treatment success. 

#### 5.2.1. Targeting Anatomy

Obesity is a key contributor to airway collapsibility. A weight reduction of 15–20% of the BMI in those with OSA significantly reduces P_crit_ (from 3.1 ± 4.2 to −2.4 ± 4.4 cm H_2_O). If P_crit_ is sufficiently reduced to below −4 cm H_2_O, there is a resolution of respiratory events [106]. Pharmacologic agents that have been shown to reduce the AHI by targeting obesity include liraglutide, semaglutide, naltrexone/bupropion, and orlistat, and by targeting fluid shifts include furosemide and spironolactone [83]. 

CPAP therapy may decrease P_crit_ by increasing both the retroglossal and retropalatal airway dimensions. A recent study of 14 participants shed light on the differences between mask interfaces from an anatomic-trait perspective. Each participant was titrated to a therapeutic pressure using both an oronasal and a nasal mask. Compared to the nasal mask, the oronasal mask was associated with a higher therapeutic CPAP requirement (+2.6 cm H_2_O ± 0.5 cm H_2_O) and higher P_crit_ (+2.4 ± 0.5 cm H_2_O) [107]. 

Oral appliance therapy reduces airway collapsibility in the retropalatal (23–29% reduction) and retroglossal (21–34% reduction) regions [108]. In a mechanistic study of 10 participants with OSA, oral appliance therapy reduced AHI scores from 25.0 ± 3.1 to 13.2 ± 4.5/h and significantly reduced P_crit_ in N2 and slow wave sleep (from −1.6 ± 0.4 to −3.9 ± 0.6 cm H_2_O and −2.5 ± 0.7 to −4.7 ± 0.6 cm H_2_O, respectively) [109]. 

Positional therapy is a treatment option for supine-predominant OSA, defined by a supine AHI that is ≥2X the non-supine AHI. Positional therapy techniques and devices can help a sleeping individual minimize time spent in the supine position. UC collapsibility (P_crit_) decreases significantly when moving from a supine to a lateral position (supine Pcrit mean 2.5 cm H_2_O, CI 1.4–3.6 to lateral PCrit mean 0.3 cm H_2_O, CI −0.8–1.4) [110], with changes that remain significant regardless of the sleep stage [111]. The change in P_crit_ from a supine to a lateral position is comparable to that with oral appliance therapy [112]. The benefits of positional therapy may not be limited to improvement in airway collapsibility. Some studies [112] (but not all) [32] also show a reduction in LG. This is consistent with observations that central sleep apnea (a condition characterized by a high LG) is less severe in a lateral compared to a supine position [113,114].

#### 5.2.2. Combination Therapy

**Figure 5:**
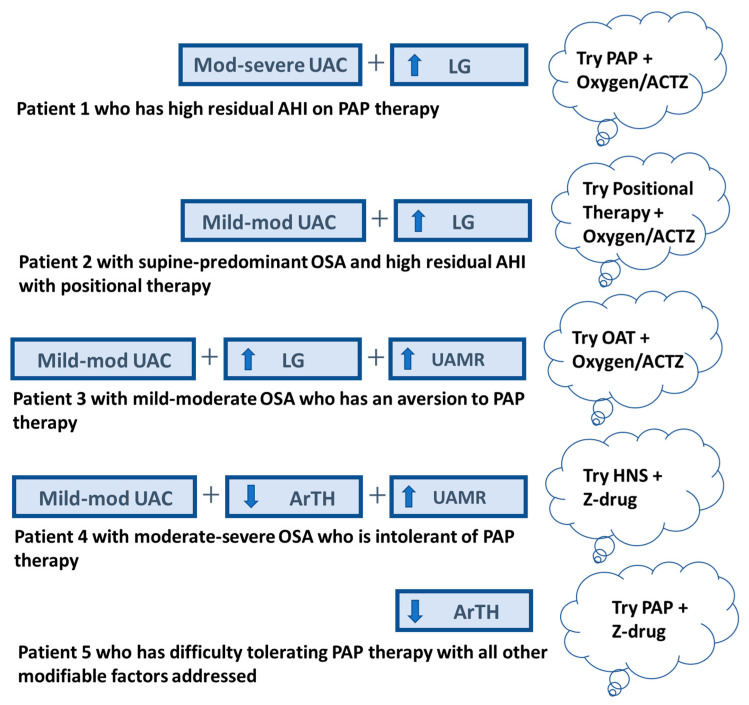
Examples of common patient scenarios in which the traits may be used to help select a “rescue” therapy. Patient 1 uses PAP therapy with good adherence, but data downloads show a high residual AHI score (e.g., AHI > 15/h). A review of this individual’s CPAP titration study shows evidence of moderate-severe UA collapsibility (UAC) (i.e., CPAP requirement > 8 cm H_2_O), and a high LG (NREM predominant OSA with “zipper” pattern on oximetry); therefore, a trial of oxygen and/or ACTZ with PAP therapy may be considered. Patient 2 is different from patient 1 in that this individual has mild-moderate UAC and supine-predominant OSA (supine AHI ≥ 2X the non-supine AHI), for which positional therapy is a potential first-line treatment. If this patient also fails positional therapy due to a high residual AHI score, oxygen and/or ACTZ can be added to positional therapy in a setting of high LG. Patient 3 is someone with mild-moderate OSA with an aversion to PAP therapy (e.g., claustrophobic with PAP) and chooses OAT. Due to a high LG and increased UAMR (see Table 2 for POC tool for LG, UAMR can be estimated using Sands et al. [58]), the predicted response to OAT alone will be inadequate; therefore, oxygen and/or ACTZ can be added. Patient 4 has moderate to severe OSA and was started on PAP therapy but ultimately discontinued therapy due to intolerance. The mild-to-moderate UAC and increased UAMR makes HNS a favorable option; however, with a low ArTH (as determined with the POC tool described in Table 2), HNS alone may not be effective at reducing the AHI. Therefore, a Z-drug can be used to raise the ArTH, in combination with HNS. Patient 5 has OSA and is using PAP therapy with difficulty tolerating therapy, frequently waking up from therapy, and is unable to keep the PAP interface on for the entire sleep duration. All other modifiable factors have been addressed (e.g., weight reduction, avoidance of alcohol, etc.). A review of the diagnostic sleep study reveals a low ArTH (see Table 2), and the addition of a Z-drug to PAP therapy can be considered. LG = loop gain; ACTZ = acetazolamide; ArTH = arousal threshold; HNS = hypoglossal nerve stimulation; OAT = oral appliance therapy; PAP = positive airway pressure; UAC = upper-airway collapsibility; UAMR = upper-airway muscle responsiveness; Z-drug = non-benzodiazepine GABA receptor agonist; up arrow = increased; down arrow = decreased.

Currently, combination therapy in OSA is considered a form of salvage treatment when the first-line therapy is inadequate. Several common clinical scenarios and potential “rescue” therapy approaches are shown in Figure 5. This approach may be promising in cases of inadequate efficacy (e.g., a high residual AHI score) or adherence, and reflects potential off-label use of some treatments (e.g., acetazolamide). To date, there are no algorithms utilizing OSA traits to address PAP failure. Notably, the approaches in Figure 5 are “off-label” and require validation in clinical trials. In addition, there are also individuals who are adherent to PAP therapy with a low residual AHI score who experience an inadequate treatment response due to persistent, excessive daytime sleepiness. The potential mechanisms of and therapy for residual hypersomnia secondary to OSA are described in detail by Javaheri et al. [115]. 

Combining UA anatomy-targeted therapies can improve efficacy. For example, the combination of positional therapy and OAT is more efficacious than either positional therapy or OAT alone [116]. The combination of CPAP therapy with oral appliances can treat OSA when OAT alone is ineffective, while reducing CPAP requirement (~9 cm H_2_O less with combined OAT and CPAP therapy than on CPAP therapy alone) [117]. Therefore, combination therapy may be a good option for those who are pressure intolerant.

Combining CPAP therapy with “z drugs” may improve adherence. A systemic review of eight studies showed an increase of 0.62 h of daily CPAP use and a 12% increase in the percentage of nights used compared to CPAP therapy alone. Eszopiclone had the most significant impact on adherence [118]. Notably, such studies include unselected patients with OSA. Some data suggest that hypnotics in those with a low ArTH might be of particular benefit [119]. A combination of a therapy to address UA collapsibility (e.g., CPAP or OAT) and a high LG (acetazolamide or oxygen) may be one way to improve the efficacy of OSA treatment [120,121].

A stepwise approach, addressing UA anatomy, followed by targeting physiological traits was examined in a study of 23 participants with OSA [122]. Participants who had a residual AHI score of >10/h with OAT alone (addressing UA anatomy) were included in a step-wise approach for additional therapy. The addition of expiratory positive airway pressure (EPAP) valve and positional therapy resulted in an AHI score of <10/h in 10 participants. A predictor of success was supine-dependent OSA. Of the remaining ten, five participants with high LG achieved therapeutic control with the addition of oxygen. Two with poor airway muscle responsiveness achieved control with atomoxetine-oxybutynin added to OAT, EPAP therapy, and positional therapy or oxygen. Of the remainder, two required CPAP therapy, and another was CPAP intolerant [122]. None of the participants qualified for the addition of a hypnotic. Three were lost to follow-up or declined further participation. While this was an exploratory study, it offers a perspective on how these traits may inform targeted combination therapy, a precision medicine approach in OSA.

### 5.3. Targeting Traits to Select Initial Therapy

Targeting the OSA traits to select the first-line therapy for each patient is the “holy grail” of physiology-based precision medicine to treat sleep apnea. For example, the combination of oxygen (lowering LG) and eszopiclone (raising the ArTH) may be effective in someone with mild UA collapsibility. In a randomized crossover study of this combination among those with an AHI score of ≥10/h, 9/20 participants responded to this combination (>50% reduction and <15/h residual AHI) in those with mild UA collapsibility [123]. Thus, in theory, carefully selecting individuals with mild-moderate UA collapsibility, high LG and a low ArTH for pharmacologic-only treatment may be viable. However, the baseline traits targeted by oxygen (LG) and eszopiclone (ArTH) in the above study did not predict treatment success, while a mild to moderately collapsible UA, and increased UA muscle responsiveness did. This highlights the complexity of targeting the OSA traits to select a first-line therapy in OSA. To date, no studies have prospectively targeted treatment based on a trait (or trait combinations) to assess effectiveness of a therapy. 

## 6. Current Limitations

Physiological traits are promising for personalizing OSA therapy. However, several critical barriers exist. First, it is unclear to what degree each trait is a cause or a consequence of OSA. Hence, in this review, we use the term “trait” (a characteristic) and not “endotype” (pathogenic mechanism). For example, a low ArTH may be a phenotype (rather than an endotype) because the ArTH is lowered with the treatment of OSA and is more common in those with lower OSA severity [42]. Thus, a low ArTH may simply serve as a biomarker of treatment effectiveness (e.g., adherence). If the traits reflect OSA’s consequences rather than causes, targeting them may not effectively eliminate UA obstruction (and may be the reason why baseline traits do not consistently predict a response to therapy targeting those traits). Second, an easy-to-use method that determines how much each trait, especially in combination with others, contributes to OSA in each individual is not yet available. Therefore, potential treatment decisions are based on arbitrary cut-offs of high versus low trait values or statistical models of a combination of traits. Such approaches are unlikely to be reproducible across patient cohorts, as prediction models in one cohort differ from models in another [77,78]. Finally, and most importantly, current studies of OSA traits and clinical outcomes are short-term, often lasting a single night, and most focus on the outcome of AHI reduction as a metric of success. Longitudinal studies examining patient-centered outcomes such as quality of life, function, and adverse consequences of OSA (e.g., endothelial function, blood pressure, neurocognition) are needed. If evidence supports targeting traits to improve patient-centered outcomes in OSA, studies assessing the implementation of a trait-based approach, including ease of use, cost, and applicability across demographic and social determinants of health, will be important.

## 7. Future Directions

Our vision for the future is for sleep providers to be able to routinely assess anatomic and non-anatomic contributors to OSA in clinical settings to (1) promote a shared decision-making process in selecting efficacious and tolerable therapy and (2) improve patient-centered outcomes. 

In order for this vision to be realized, additional physiologic research as well as implementation science work are needed to address both the use of traits to predict treatment responses and to target traits for a more precise treatment approach. Below are the key challenges to be addressed:

(1) Standardized, reliable, and reproducible tools must be developed to measure the OSA traits using readily available clinical data (e.g., home respiratory polygraphy, wearables). Such tools should integrate how much each trait, or trait combination, contributes to OSA severity in each individual.
(2) A better understanding is required of how traits relate to clinical phenotypes (e.g., the ArTH and LG in those with OSA and insomnia) and their contributions to OSA in those with common, co-morbid conditions (e.g., post-traumatic stress disorder, chronic obstructive pulmonary disease, opioid dependence).
(3) Studies are needed to determine the effect sizes of interventions targeting the OSA traits (e.g., acetazolamide for high LG) and their impact on OSA severity.
(4) Prospective validation of the traits (or their combinations) is needed to predict treatment responses to established therapies (e.g., randomization to a sedative-hypnotic vs. a placebo with CPAP based on the ArTH to assess the impact on CPAP adherence, daytime function, and quality of life).
(5) Longer-term (at least 3–6 months), randomized clinical trial studies on patient-centered outcomes are needed, for both prediction of outcomes and modification of the OSA traits to improve OSA outcomes. Studies should examine prognostic markers that are more effective than the AHI in assessing OSA alleviation (e.g., hypoxic burden, heart rate response), adherence, patient symptoms, function, and quality of life.
(6) Because little is known about the role of OSA traits in patient outcomes in non-white and non-male individuals, an assessment of traits in pathogenesis, clinical manifestations, and treatment outcomes in non-white and female populations is needed.

## 8. Summary 

The interplay of the four key OSA traits, including UA airway collapsibility capturing anatomical predisposition and the remaining physiological traits of LG, ArTH, and upper-airway muscle responsiveness, can result in different pathways to OSA among individuals. These unique combinations offer a potential approach for precision medicine approach in OSA. For example, retrospective studies demonstrate that the traits predict treatment response of established therapies for OSA, including CPAP therapy, oral appliances, and hypoglossal nerve stimulation. Recent advances in the field show that these traits can be estimated from clinical data, including signal processing of polysomnography or even simple event-count-per-hour metrics such as the AHI, the fraction of hypopneas and the oxygen saturation nadir (Table 2). While not yet ready for “prime time”, using these traits to select multi-modal rescue therapies for those failed by CPAP therapy can be a significant step towards improving the “one-size fits all” CPAP approach. The ultimate goal is to use these traits to select initial treatment. For now, the focus of the OSA field should include developing scalable and reliable methods for their assessment and longitudinal patient-centered validation of the OSA traits’ utility for therapy selection (see Table 2).

## Figures and Tables

**Table 1:** Key points.

The collapsibility of the upper airway, largely driven by anatomy, is a key determinant of OSA risk.
When anatomic predisposition alone is not sufficient to cause OSA, physiologic traits such as a low arousal threshold (ArTH), high loop gain (LG), and poor upper airway muscle response (UAMR) can lead to OSA (Figure 1 and Figure 2).
Retrospective analyses show that these traits can predict a response to current OSA treatments (Figure 3). For example, a high ArTH, low LG, and UAMR predict the success of oral appliance therapy.
Measurements of the traits from clinical polysomnography require signal processing expertise and specialized arousal scoring. Point-of-care of care methods to estimate the traits exist (Table 2) but require prospective validation.
Characterizing and targeting OSA traits that are failed by initial OSA therapy (e.g., CPAP) may offer one avenue to “rescue” treatments (Figure 4).
Before evidence-based clinical use of traits to tailor therapy for OSA, several steps are critical, including the following: (a) standardized and reliable methods of trait measurement, (b) prospective, long-term (over 6 months) validation of the trait’s utility in predicting outcomes of established OSA therapies (e.g., CPAP, oral appliances), (c) prospective trials selecting first line therapy for each individual based on OSA traits, and (d) once the above are established, implementation studies focused on ease of use, cost and applicability to diverse populations are required (see Section 6 and Section 7).

**Table 2:** Summary of Point of Care Clinical Tools for Measuring Traits.

**Upper Airway Collapsibility**: A CPAP requirement of ≤8 cm H_2_O on titration PSG predicts a mildly collapsible airway (89% sensitive and 84% specific) [62].
**Arousal Threshold**: A point is given for each of the following: AHI < 30 events per hour, nadir oxygen saturation > 82.5%, and the fraction of the AHI that are hypopneas (F_hypopneas_) > 58.3%. A score of 2 or more predicts a low ArTH (80% sensitive and 88% specific) [65].
**Loop Gain**: (1) LG = 2π/[2πDR − sin(2πDR)] on titration PSG with the presence of treatment-emergent central sleep apnea (TE-CSA) where DR is the ratio of the duration of the ventilatory phase (time from the end of one apnea to the start of the next) to the total cycle duration (time from the end of one apnea to the end of the next) [64]. (2) A “zipper-like” pattern of oxygen desaturation on hypnograms in NREM-predominant OSA suggest high LG [67,68].
**Upper Airway Muscle Responsiveness**: No published POC tool exist. A tool developed by Sands et al. [58] can be used to estimate UAMR and other traits from clinical PSG.

## Data Availability

Not applicable.
